# Prognosis of severe lymphopenia after postoperative radiotherapy in non-small cell lung cancer: Results of a long-term follow up study

**DOI:** 10.1016/j.ctro.2021.02.011

**Published:** 2021-03-12

**Authors:** Wang Jing, Yufei Liu, Hui Zhu, James Welsh, Saumil Gandhi, Melenda Jeter, Quynh Nguyen, Aileen B. Chen, Michael O'Reilly, Zhongxing Liao, Joe Y. Chang, Percy Lee, Steven H. Lin

**Affiliations:** a Department of Radiation Oncology, The University of Texas MD Anderson Cancer Center, Houston, TX 77030, USA; b Department of Radiation Oncology, Shandong Cancer Hospital and Institute, Shandong First Medical University and Shandong Academy of Medical Science, Shandong, China

**Keywords:** Non-small cell lung cancer, Lymphopenia, Chemoradiation, Postoperative radiotherapy, Prognosis

## Abstract

**Purpose:**

To investigate the incidence and prognosis of severe radiation-induced lymphopenia (sRIL) after postoperative radiotherapy (PORT) for resected NSCLC.

**Patients and methods:**

Between 1998 and 2017, 170 patients treated with PORT for NSCLC were retrospectively reviewed. Lymphopenia was divided into tertiles with severe lymphopenia defined as absolute lymphocyte counts (ALC) < 0.37 × 10^3^/ul.

**Results:**

sRIL was observed in 32.3% of patients. Multivariable logistic regression analysis indicated sRIL was associated with planning target volume radiation fraction numbers (OR 1.09, *p* = 0.005) and total lung mean dose (OR 1.12, *p* = 0.006). With a median follow-up time of 12.2 years, the median progression-free survival and overall survival were 14.8 months and 28.4 months respectively in patients with sRIL, vs. 21.7 months (*p* = 0.008) and 48.3 months (*p* = 0.01) respectively in patients without sRIL. Multivariable analyses indicated sRIL significantly decreased OS (HR 1.95, *p* < 0.01). Since PORT for stage I-II NSCLC was done largely for positive margins, which may confound the contribution of severe RIL, we analyzed stage III separately and found that sRIL also significantly decreased OS (HR 1.88, *p* = 0.004) in multivariable analysis.

**Conclusion:**

For this long-term outcome study, severe RIL correlated with total lung mean dose and radiation fractionation numbers, and was a strong prognostic factor for poor survival in PORT patients, particularly in patients with stage III NSCLC, highlighting the importance of an intact immune system for post-radiation immunologic disease surveillance.

## Highlights

• First study to investigate lymphopenia in NSCLC with postoperative radiotherapy.
• Radiation-induced lymphopenia is common is this population.
• Lymphopenia is correlated with radiation fraction number and total mean lung dose.
• Lymphopenia is an independent risk factor for PFS and OS, particularly in stage III NSCLC.

## Introduction

The role of postoperative radiation (PORT) in resectable non-small cell lung cancer (NSCLC) remains controversial, particularly in stage III disease [1], [2]. The ANITA trial that indicated that patients with pN2 disease could benefit from PORT but patients with pN1 disease did not [3]. However, 95% of patients with clinical stage III-N2 disease receiving PORT were with distant recurrence while only 5% developed an initial isolated local recurrence [2]. Overall survival (OS) was only 43.7% at 5 years, while the locoregional failure-free survival and distant metastasis-free survival were 53.4% and 42.5%, respectively [4].

Radiotherapy was traditionally thought to be solely a local therapy, but the consensus in the era of immunotherapy is that radiation has systemic effects on the host’s immune system. Lymphopenia is a common treatment-related toxicity in cancer patients undergoing radiotherapy. Lymphocytes are highly radiosensitive with their numbers decreasing significantly after radiation and remaining at a low level even for months after CRT [5], [6]. For patients treated with chemoradiation for NSCLC, approximately 43% of patients developed grade 3 or 4 lymphopenia after radiation [7]. The incidence of grade ≥3 lymphopenia increased to 87% after concurrent chemoradiation [8]. Furthermore, emerging evidence indicated that lymphopenia was correlated with inferior survival in several solid tumors such as hepatocellular carcinoma, glioma, esophageal cancer, nasopharyngeal carcinoma, as well as lung cancer [9], [10], [11], [12], [13]. Grade ≥3 lymphopenia was found to be a negative factor for OS (HR 1.5, *p* = 0.01) in patients receiving definitive chemoradiation for stage III NSCLC [8]. Therefore, determining the effectors of lymphopenia is crucial for further mitigation strategies to protect the host’s immune system, which may translate into survival benefits.

Numerous studies have investigated the factors affecting the degree of lymphopenia. In esophageal cancer, proton therapy was superior compared to photon therapy in mitigating lymphopenia [14]. Older age, lower tumor location, greater tumor length, and larger planning target volume (PTV) exacerbated lymphopenia [15]. The nomogram developed in esophageal cancer indicated age, PTV in interaction with body mass index (BMI), radiation technique, and baseline absolute lymphocyte count (ALC) were factors associated with treatment-related lymphopenia [16]. A study in limited-stage small cell lung cancer revealed that patterns of radiation fractionation also affect lymphopenia [17]. In NSCLC patients receiving definitive radiation, larger gross tumor volumes correlated with lower lymphocytes nadirs after radiotherapy [18].

The role of lymphopenia in patients with PORT has not been well studied. Therefore, we conduct a retrospective study to investigate the relationship of patients’ characteristics and radiation-related parameters, as well as baseline ALC, with the risk of severe radiation-induced lymphopenia (sRIL) during CRT in NSCLC patients treated with PORT. The secondary aim was to assess the predictive value of sRIL for clinical outcomes.

## Patients and methods

### Patients

This is an Institutional Review Board approved cohort study in lung cancer patient treated with radiotherapy. Between 1998 and 2017, the medical records from all consecutive patients who underwent surgery followed by radiation (with or without chemotherapy) for NSCLC were extracted. Inclusion criteria were patients who received radiation after surgery who had availability of complete blood count and follow-up data, Eastern Cooperative Oncology Group performance status ≥2, and no induction chemotherapy. Potential predictors of lymphopenia were recorded, including gender, age, race, BMI, medical complication, smoking status, tumor histology, differentiation grade, tumor location, tumor size, pathological T- and N-stage, pathological stage, and RT-related parameters, such as PTV, radiation modality, total radiation dose (to PTV), fraction number, and lung/heart radiation parameters. Heart or lung V5 was defined as heart or total lung minus-PTV relative percent volumes receiving 5 Gy. All patients were restaged from stage I to III according to the American Joint Committee on Cancer version 7. Patients who underwent interrupted radiotherapy were excluded.

### Treatment approaches

All patients treated with PORT by either photon therapy or proton therapy were included. Three-dimensional conformal radiotherapy (3D-CRT), intensity-modulated radiation therapy (IMRT), and volumetric-modulated arc therapy (VMAT) were categorized as photon therapy, while intensity-modulated proton therapy and passive scattering proton therapy were categorized as proton therapy. Two-dimensional radiation was not used in this study. Patients received 50–64 Gy in 25–33 fractions with 1.8 Gy–2.0 Gy per fractionation. In addition, patients who received hyperfractionated radiation twice daily with 1.2 Gy were also eligible. Platinum-based chemotherapy was given peri radiotherapy.

### Absolute lymphocyte count assessment

The values of ALC were collected at pre-radiation, during RT, and after 1-month post RT (if applicable). Due to only 16 patients having grade 4 lymphopenia according to the Common Terminology Criteria for Adverse Events version 5.0, lymphopenia was divided into tertiles for the entire population, with sRIL defined as the lower tertile value as absolute lymphocyte counts (ALC) < 0.37 × 10^3^/ul to minimize the analysis errors.

### Statistical analysis

Categorical variables were summarized by frequencies and percentages and compared between the two groups with Chi-square tests or Fisher’s exact tests; continuous variables were summarized using means, standard deviations, medians, and ranges and assessed between groups by two-sample t-tests or Wilcoxon rank-sum tests (Kruskal-Wallis tests/ANOVA if appropriate). Generalized linear regressions were used to evaluate the associations between lymphopenia (sRIL versus non-sRIL) and covariates of interest. Unadjusted survival distributions were estimated by the Kaplan-Meier method, and comparisons were made with the log-rank test. Cox proportional hazards regression models were used to evaluate the associations between survival outcomes and covariates of interest. All statistical analyses were performed using SPSS version 23.0 (IBM Corp., NY, USA) with 0.05 as a significance level.

## Results

### Patients

We analyzed a total of 170 patients treated with PORT for NSCLC. The median age for the whole cohort was 62 years (range, 32–89 years). The majority of patients were male (55.3%), white (79.4%), prior/current smoker (83.5%), had adenocarcinoma histology (60.0%), had N2 disease (65.3%) and were clinical stage III (74.1%). Of the 126 patients with stage III disease, 87.3% of patients had N2 disease, while 88.9% were stage IIIA. The median tumor size was 3.9 cm (range, 0.8–14.5 cm). Lobectomy was performed in 79.4% of patients, and 68.8% of patients had a complete resection (R0). 63.5% of patients were given postoperative chemotherapy (POCT) with a median of 4 cycles (range, 1–8). The clinical characteristics are summarized in Table 1.

**Table 1:** Clinical characteristics of patient with severe radiation-induced lymphopenia (sRIL) and non-sRIL.

Characteristics	No. (%) n = 170	s-RIL (%) (n = 55)	Non-sRIL (%) (n = 115)	*p* value
Gender				
Male	94 (55.3)	36 (65.5)	58 (50.4)	0.07
Female	76 (44.7)	19 (34.5)	57 (49.6)	
Race				
White	135 (79.4)	45 (81.8)	90 (78.3)	0.69
Non-White	35 (20.6)	10 (18.2)	25 (21.7)	
Age (Mean ± SD)				
≥60	107 (62.9)	33 (60.0)	74 (64.3)	0.61
<60	63 (37.1)	22 (40.0)	41(35.7)	
CardioDis				
No	143 (84.1)	49 (89.1)	94 (81.7)	0.27
Yes	27 (15.9)	6 (10.9)	21 (18.3)	
COPD				
No	145 (85.3)	45 (81.8)	100 (87.0)	0.49
Yes	25 (14.7)	10 (18.2)	15 (13.0)	
Smoking				
Prior/current	142 (83.5)	48 (87.3)	94 (81.7)	0.39
Never	28 (16.5)	7 (12.7)	21 (18.3)	
Px tumor location				
Right lung	97 (57.1)	27 (49.1)	70 (60.9)	0.18
Left lung	73 (42.9)	28 (50.9)	45 (39.1)	
Surgery				
Sublobar resection	19 (11.2)	7 (12.7)	12 (10.4)	0.78
Lobectomy	135 (79.4)	42 (76.4)	93 (80.9)	
Pneumonectomy	16 (9.4)	6 (10.9)	10 (8.7)	
Pathological type				
ADC	102 (60.0)	31 (56.4)	71 (61.7)	0.78
SCC	50 (29.4)	18 (32.7)	32 (27.8)	
NEU	18 (10.6)	6 (10.9)	12 (10.4)	
LVI				
Yes	61 (39.6)	16 (31.4)	45 (43.7)	0.16
No	93 (60.4)	35 (68.6)	58 (56.3)	
Differentiation grade				
Well	13 (7.6)	4 (8.2)	9 (8.9)	0.96
Moderate	73 (42.9)	23 (46.9)	50 (49.5)	
Poor	64 (37.6)	22 (44.9)	42 (41.6)	
Surgical margin				
R0	117 (68.8)	30 (54.5)	87 (75.7)	0.01
R1/2	53 (31.2)	25 (45.5)	28 (24.3)	
pT stage				
T1–2	119 (70.0)	35 (63.6)	84 (73.0)	0.28
T3–4	51 (30.0)	20 (36.4)	31 (27.0)	
pN stage				
N0–1	56 (32.9)	23 (41.8)	33 (28.7)	0.12
N2–3	114 (67.1)	32 (58.2)	82 (71.3)	
pStage				
I–II	43 (25.3)	17 (30.9)	26 (22.6)	0.26
III	127 (74.7)	38 (69.1)	89 (77.4)	
Adjuvant Chemo				
Yes	108 (63.5)	34 (61.8)	74 (64.3)	0.86
No	61 (35.9)	21 (38.2)	41 (35.7)	
RT technique				
Photon	154 (90.6)	51 (92.7)	103 (89.6)	0.59
3D-CRT	100 (64.9)	34 (22.1)	66 (42.8)	
IMRT	43 (27.9)	17 (11.0)	26 (16.9)	
VMAT	11 (7.1)	0 (0.0)	11 (7.1)	
Proton	16 (9.4)	4 (7.3)	12 (10.4)	
BMI		25.4 ± 5.07	26.2 ± 4.17	0.34
Tumor size		4.80 ± 2.71	4.11 ± 2.33	0.09
Baseline ALC		1.63 ± 0.66	1.65 ± 0.74	0.81
PTV (mean ± SD, cm^3^)		475.6 ± 303.3	350.9 ± 214.1	0.015
Median PTV dose (range)		58.4 ± 6.9	54.4 ± 5.4	<0.001
Median RTfxNo. (range)		32.2 ± 7.4	28.5 ± 5.7	<0.001

*Abbreviations:* CardioDis, Cardiovascular disease; COPD, chronic obstructive pulmonary disease; Px, primary; ADC, adenocarcinoma; SCC, squamous cell carcinoma; NEU, neuroendocrine carcinoma; R0/R1/R2: complete resection, microscopic residual tumor, macroscopic residual tumor; LVI, lymphovascular invasion; pT/N stage, pathological tumor/node stage; RT, radiation; ALC, absolute lymphocyte count. 3D-CRT, three-dimensional conformal radiation therapy; IMRT, Intensity-modulated radiation therapy; VMAT, Volumetric modulated arc therapy; BMI, body mass index; PTV, planning targeted volume; RTfxNo., radiation fraction number.

As shown in Table 1, photon therapy (90.6%) was the dominant RT technique used. The median PTV dose was 54 Gy (range, 48.6–64 Gy) in the whole group, while it was 52 Gy in the sRIL group and 50.4 Gy in the non-sRIL group. For the whole cohort, 36.5% and 22.2% of patients were given 50 Gy and 60 Gy, respectively, while it was 47.0% and 20.9% in non-sRIL, respectively. In sRIL population, 14.5% of patients were given 50 Gy, 50.4 Gy and 63 Gy, respectively. For the dose per fraction in the sRIL group, 70.6% and 22.4% of patients were treated in 2.0 Gy and 1.8 Gy, respectively, while it was 78.3% and 15.7% in the non-sRIL group, respectively.

### Lymphopenia during treatment and associated factors

The median interval from the date of surgery to radiation was 1.8 months (IQR, 1.4–2.4), while it was 1.8 months to chemotherapy (IQR, 1.4–3.1). The median interval from surgery to the first time to collect lymphocytes was 1.6 months (IQR, 1.2–2.3). In addition, the median interval between POCT and PORT was 0.8 months (IQR, 0.0–2.5). After surgery but before PORT, the median of ALC for the whole cohort is 1.52 × 10^3^/ul with ranging from 0.45 − 4.35 × 10^3^/ul. In addition, before PORT, a total of 23 (13.5%) patients experienced lymphopenia, while 11 patients with grade 1, 10 patients with grade 2, and 2 patients with grade 3, but none of them had sRIL as defined by ALC < 0.37 × 10^3^/ul. Among these 23 patients, 7 patients developed sRIL in the period of RT, while 2 experienced grade 4 lymphopenia. In the whole groups, a total of 55 (32.3%) patients experienced sRIL during RT while 115 patients had non-sRIL. The median of ALC for the 55 patients was 0.26 × 10^3^/ul (range, 0.08–0.36 × 10^3^/ul). Of the patients with stage III disease, 29.9% of patients experienced sRIL. Clinical features were well-balanced between the two groups except for surgical margins with significantly more patients with R1/2 (45.5%) in sRIL compared to non-sRIL (24.3%) (*p* = 0.01).

The dosimetric variables were further investigated between the sRIL and non-sRIL groups. As shown in Table 1, the mean PTV and PTV dose in sRIL were 475.6 cm^3^ and 58.4 Gy, which were significantly higher than 350.9 cm^3^ and 54.5 Gy in non-sRIL (*p* = 0.015, and *p* < 0.001). Moreover, the median radiation fraction numbers were also higher in patients with sRIL (32.2 vs. 28.5, *p* < 0.001). In addition, the mean RT dose, V5, V10, and V20 of total lung were also significantly higher in patients with sRIL (Supplementary Table 1).

The correlation between the clinical characteristics, radiation-related parameters, and lymphopenia was further explored to identify the potential predictors of sRIL. As shown in Table 2, only surgical margin status (HR 2.59, *p* = 0.01), radiation fractionation (HR 1.09, *p* < 0.01), fraction size (HR 0.32, *p* < 0.01), total lung mean RT dose (HR 1.10, *p* = 0.01), total lung V10 (HR 1.03, *p* = 0.04), and total lung V20 (HR 1.04, *p* = 0.03) were correlated with sRIL in univariable logistic regression analysis. In multivariable logistic regression analysis, only gender (HR 2.38, *p* = 0.036), radiation fractionation numbers (HR 1.09, *p* = 0.005), and total lung mean dose (HR 1.12, *p* = 0.006) were associated with sRIL.

**Table 2:** Univariate and multivariate analyses for association with severe radiation-induced lymphopenia.

Characteristic	Univariate		Multivariate	
	OR (95% CI)	*p*	OR (95% CI)	*p*
Gender (Male vs. Female)	1.86 (0.96–3.62)	0.07	2.38 (1.06–5.36)	0.036
Race (White vs. Non-white)	1.25 (0.55–2.83)	0.59		
Age (≥60 vs. <60)	0.83 (0.43–1.61)	0.58		
BMI	0.96 (0.87–1.05)	0.34		
CardioDis	0.55 (0.21–1.45)	0.22		
COPD	1.48 (0.62–3.55)	0.38		
Smoking^a^	1.53 (0.61–3.86)	0.36		
Tumor location				
Right vs. Left	0.62 (0.32–1.18)	0.15		
Surgery type				
Sublobar resection	Ref			
Lobectomy	0.77 (0.28–2.11)	0.62		
Pneumonectomy	1.03 (0.26–4.07)	0.97		
Pathological type				
NEU	Ref			
ADC	0.87 (0.30–2.54)	0.80		
SCC	1.12 (0.36–3.51)	0.84		
Tumor grade				
Well	Ref			
Moderate	1.03 (0.29–3.71)	0.96		
Poor	1.18 (0.33–4.26)	0.80		
Surgical margin				
R1/2 vs. R0	2.59 (1.31–5.11)	0.01		
Tumor size	1.12 (0.98–1.27)	0.10		
pT-stage				
T3–4 vs. T1–2	1.55 (0.78–3.08)	0.21		
pN-stage				
N2–3 vs. N1–2	0.56 (0.29–1.09)	0.09		
pStage				
III vs. I–II	0.69 (0.33–1.40)	0.30		
AdjChemo	0.90 (0.46–1.74)	0.75		
RT modality				
Proton vs. Photon	0.67 (0.21–2.19)	0.51		
Photon therapy				
3D-CRT	Ref			
IMRT*	0.87 (0.43–1.76)	0.70		
RTfxNo.	1.09 (1.03–1.15)	<0.01	1.09 (1.03–1.17)	0.005
RTfxSize (2.0 Gy vs. 1.8 Gy)	0.32 (0.16–0.63)	<0.01		
PTV	1.00 (0.98–1.03)	0.01		
Heart V5	1.00 (0.99–1.02)	0.41		
Heart V10	1.01 (1.00–1.02)	0.20		
Heart V20	1.01 (0.99–1.02)	0.27		
Heart V30	1.01 (1.00–1.03)	0.19		
Heart V40	1.01 (0.99–1.03)	0.24		
Heart V50	1.02 (0.99–1.05)	0.16		
Heart mDose	1.01 (0.98–1.02)	0.28		
Total lung mDose	1.10 (1.02–1.18)	0.01	1.12 (1.03–1.21)	0.006
Total lung V5	1.02 (1.00–1.04)	0.05		
Total lung V10	1.03 (1.00–1.06)	0.04		
Total lung V15	1.03 (1.00–1.06)	0.07		
Total lung V20	1.04 (1.01–1.08)	0.03		
Baseline ALC	0.94 (0.59–1.50)	0.81		

*Abbreviations:* BMI, body mass index; COPD, chronic obstructive pulmonary disease; Smoking^a^, prior/current vs. never; pT/N stage, pathological tumor/node stage; AdjChemo, adjuvant chemotherapy; PTV, planning target volume; RT, radiation; 3D-CRT, three-dimensional conformal radiation therapy; IMRT*, Intensity-modulated radiation therapy (including VMAT, Volumetric modulated arc therapy); RTfxNo., radiation fraction number; V5, organ volume receiving 5 Gy; mDose, mean radiation dose; ALC, absolute lymphocyte count.

### Lymphopenia and survival outcomes

**Fig. 1:**
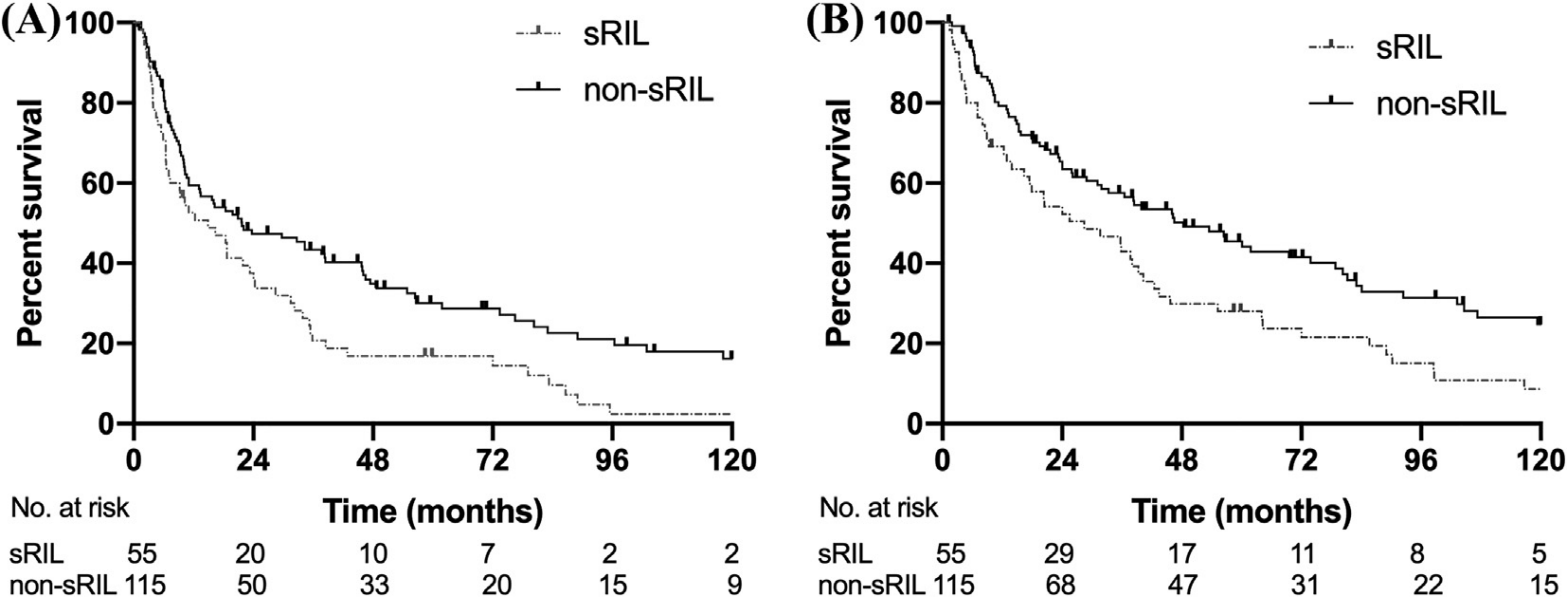
Progression-free survival (PFS) and Overall survival (OS) of all the patients with or without severe radiation-induced lymphopenia. (A) The median PFS in sRIL is 14.9 months versus 21.7 months in non-sRIL (*p* = 0.008). (B) The median OS in sRIL is 28.4 months, compared to 48.3 months in non-sRIL (*p* = 0.01).

The median follow-up for the cohort was 12.2 years (interquartile range, 4.7–14.6 years). 25.9% (44/170) of patients were alive at last follow up. The median PFS and OS for the whole group were 19.8 months and 38.4 months, respectively (Supplementary Fig. 1). For patients with sRIL, the median PFS was 14.9 months, whereas it was 21.7 months in the non-sRIL group (*p* = 0.008, Fig. 1A). For patients with sRIL, the median OS was 28.4 months, which was significantly worse than the 48.3 months in patients with non-sRIL (*p* = 0.01, Fig. 1B). The 1-, 3-, and 5-year survival rates in the sRIL group were 69.1%, 42.9%, and 28.0%, respectively, in contrast to 79.3%, 57.6%, and 45.5% in the non-sRIL group, respectively.

Univariable analysis indicated that gender, pathological stage, and lymphopenia were correlated with PFS for the whole cohort (Table 3). Multivariable analysis revealed that pathological stage (HR 1.61, *p* = 0.019) and sRIL (HR 1.68, *p* = 0.004) were independent predictors of PFS.

**Table 3:** Univariate and multivariate analyses for progression-free survival in the whole group.

Characteristic	Univariable analysis		Multivariable analysis	
	HR (95%CI)	*p*	HR (95%CI)	*p*
Gender				
Male vs. Female	1.49 (1.06–2.10)	0.02		
Race				
White vs. Non-white	1.25 (0.80–1.94)	0.32		
Age (≥60 vs. < 60)	0.95 (0.67–1.34)	0.76		
CardioDis				
COPD	1.26 (0.80–2.00)	0.32		
Smoking^a^	1.43 (0.89–2.27)	0.13		
Tumor site				
Right vs. Left	0.85 (0.61–1.20)	0.37		
Surgery type				
Sublobar resection	Ref			
Lobectomy	1.00 (0.58–1.73)	0.99		
Pneumonectomy	1.40 (0.68–2.87)	0.36		
Pathological type				
NEU	Ref			
ADC	1.08 (0.63–1.86)	0.77		
SCC	1.49 (0.84–2.66)	0.17		
Surgical margin				
R1/2 vs. R0	1.14 (0.79–1.63)	0.49		
LVI	1.27 (0.88–1.83)	0.19		
Tumor grade				
Well	Ref			
Moderate	0.71 (0.37–1.36)	0.30		
Poor	0.86 (0.45–1.65)	0.65		
pT stage				
T3–4 vs. T1–2	1.17 (0.81–1.70)	0.40		
pN stage				
N2–3 vs. N0–1	1.26 (0.88–1.82)	0.21		
pStage (III vs. I–II)	1.52 (1.02–2.26)	0.04	1.61 (1.08–2.40)	0.019
Adjuvant Chemo	0.79 (0.56–1.11)	0.18		
RT technique				
Proton vs. Photon	1.02 (0.56–1.85)	0.94		
Lymphopenia				
sRIL vs. non-sRIL	1.60 (1.13–2.27)	0.01	1.68 (1.18–2.39)	0.004

*Abbreviations:* CardioDis, Cardiovascular disease; COPD, chronic obstructive pulmonary disease; Smoking^a^, Prior/Current vs. Never; NEU, neuroendocrine carcinoma; ADC, adenocarcinoma; SCC, squamous cell carcinoma; R0/R1/R2: complete resection, microscopic residual tumor, macroscopic residual tumor; LVI, lymphovascular invasion; pT/N stage, pathological tumor/node stage. sRIL, severe radiation-induced lymphopenia.

As shown in Table 4, squamous cell histology, adjuvant chemotherapy, and lymphopenia were correlated with OS in the univariable analysis. Multivariable analysis demonstrated that sRIL (HR 1.95, *p* < 0.01), squamous cell histology (HR 2.39, *p* = 0.002), pathological stage (HR 2.01, *p* = 0.002), and adjuvant chemotherapy (HR 0.60, *p* = 0.006) were independent predictors of OS.

**Table 4:** Univariate and multivariate analyses for overall survival in the whole group.

Characteristic	Univariable analysis		Multivariable analysis	
	HR (95% CI)	*p*	HR (95% CI)	*p*
Gender				
Male vs. Female	1.41 (0.85–2.17)	0.06		
Race				
White vs. Non-white	1.36 (0.80–2.17)	0.20		
Age (≥60 vs. <60)	1.28 (0.89–1.84)	0.19		
CardioDis	1.19 (0.74–1.90)	0.47		
COPD	1.35 (0.83–2.18)	0.22		
Smoking^a^	1.63 (0.98–2.73)	0.06		
Tumor site				
Right vs. Left	1.00 (0.70–1.43)	0.99		
Surgery type				
Sublobar resection	Ref			
Lobectomy	1.16 (0.66–2.03)	0.60		
Pneumonectomy	1.09 (0.50–2.36)	0.83		
Pathological type				
Neu	Ref		Ref	
ADC	1.39	0.30	1.75 (0.94–3.25)	0.079
SCC	2.08	0.03	2.93 (1.49–5.76)	0.002
Surgical margin				
R1/2 vs. R0	1.26 (0.88–1.84)	0.22		
LVI	1.10 (0.75–1.62)	0.61		
Tumor grade				
Well	Ref			
Moderate	0.74 (0.37–1.45)	0.37		
Poor	0.88 (0.45–1.74)	0.71		
pT stage				
T3–4 vs. T1–2	1.30 (0.88–1.92)	0.19		
pN stage				
N2–3 vs. N0–1	1.15 (0.79–1.67)	0.47		
pStage (III vs. I–II)	1.41 (0.94–2.13)	0.10	2.01 (1.29–3.13)	0.002
AdjChemo	0.67 (0.47–0.96)	0.03	0.60 (0.42–0.86)	0.006
RT technique				
Proton vs. Photon	0.98 (0.50–1.93)	0.95		
Lymphopenia				
sRIL vs. non-sRIL	1.60 (1.12–2.28)	0.01	1.95 (1.34–2.82)	<0.01
BMI	1.01 (0.96–1.06)	0.74		
Baseline ALC	1.19 (0.92–1.55)	0.19		

*Abbreviations:* CardioDis, Cardiovascular disease; COPD, chronic obstructive pulmonary disease; Smoking^a^, Prior/Current vs. Never; NEU, neuroendocrine carcinoma; ADC, adenocarcinoma; SCC, squamous cell carcinoma; R0/R1/R2: complete resection, microscopic residual tumor, macroscopic residual tumor; LVI, lymphovascular invasion; pT/N stage, pathological tumor/node stage; POCT, postoperative chemotherapy. BMI, body-mass index; ALC, absolute lymphocyte counts.

### Subgroup analysis in patients with stage III NSCLC

**Fig. 2:**
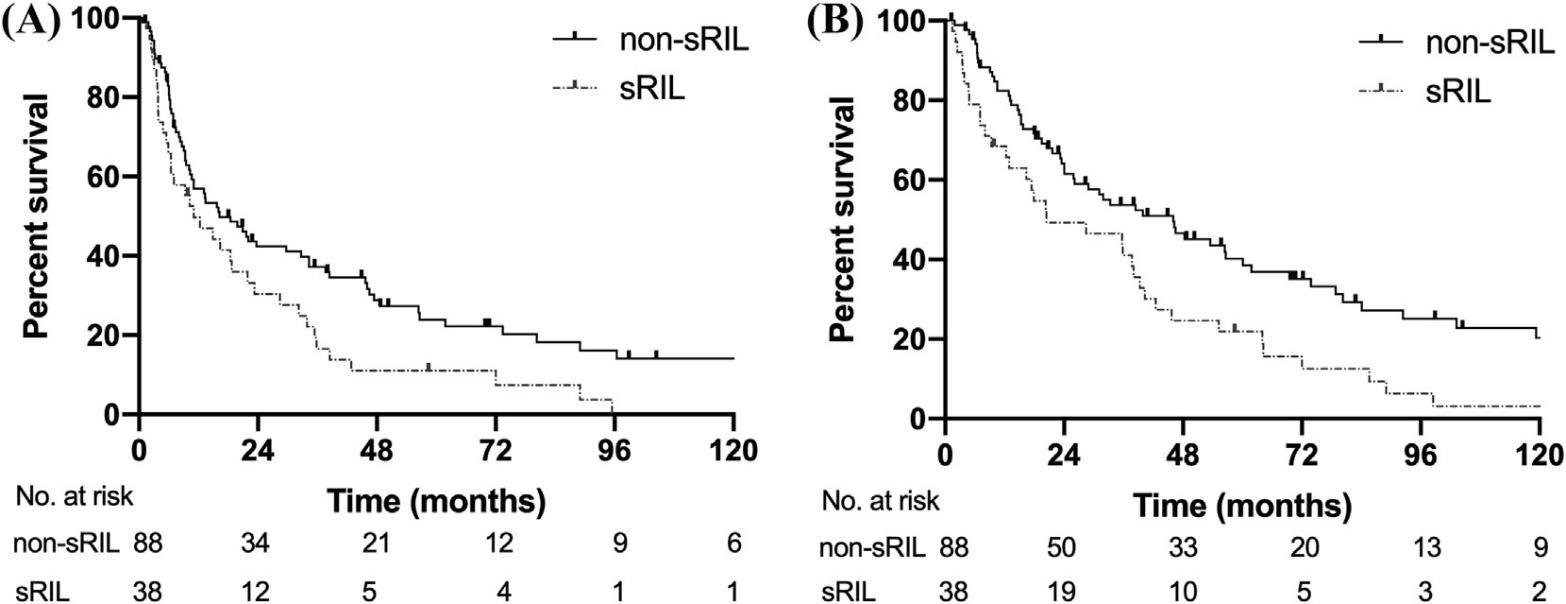
Progression-free survival (PFS) and overall survival (OS) in stage III NSCLC patient with or without sRIL. (A) The median PFS of sRIL was 11 months vs. 18.4 months in non-sRIL (*p* = 0.015); (B) The median OS in sRIL was 20.4 months, compared to 46.0 months in non-sRIL (*p* = 0.006).

Since PORT given to stage I-II patients is mostly due to close or positive surgical margins which is a competing prognostic risk compared to sRIL, we performed a subgroup analysis only in stage III patients. For patients with sRIL, the median PFS was 11 months vs. 18.4 months in patients with non-sRIL (*p* = 0.015; Fig. 2A). Similarly, the median OS was worse in the sRIL group (20.4 vs. 46.0 months, *p* = 0.006; Fig. 2B).

In univariable analysis, smoking status (*p* = 0.02), squamous cell histology (*p* = 0.04), pT stage (*p* = 0.04), and lymphopenia (*p* = 0.02) were associated with PFS (Supplementary Table 2). Only smoking status (HR 1.79, *p* = 0.026) and sRIL (HR1.62, *p* = 0.021) were predictive factors on multivariable analysis. Race (*p* = 0.04), smoking status (*p* = 0.01), squamous cell histology (*p* = 0.01), pN stage (*p* = 0.01), and lymphopenia (*p* = 0.01) were associated with OS in univariable analysis (Supplementary Table 3). Multivariable analysis revealed that race (HR 1.78, *p* = 0.037), squamous cell histology (HR 3.15, *p* = 0.002), and sRIL (HR 1.88, *p* = 0.004) were independent predictors of OS.

## Discussion

In the present study, we investigated the factors associated with lymphopenia in patients receiving PORT. Lymphopenia occurred in all patients with 32.3% of patients having sRIL. sRIL was associated with gender, radiation fractionation, and total lung mean RT dose. Moreover, sRIL was significantly correlated with decreased PFS and OS. On a subgroup analysis of stage III patients, sRIL was also associated with poorer survival. Therefore, sRIL appears to be strongly prognostic for PFS and OS in patients with PORT. These findings indicate that lymphopenia is very common in this population and has a negative impact on outcomes. These results suggest that modifications in radiation treatment parameters may mitigate lymphopenia and improve clinical outcomes.

Radiation-associated lymphopenia has been investigated in several studies. Huang et al. showed that severe lymphopenia was correlated with female, old age, lower baseline total lymphocyte count, and higher brain volume receiving 25 Gy in high-grade glioma with radiation plus temozolomide [10]. Susannah et al. showed that RT field size, dose per fraction, and fraction number were correlated with lymphopenia [19]. Our study is consistent with the view that the extent of radiation exposure, regardless of context, is lymphocyte depleting. Increased fraction numbers were associated with sRIL (HR 1.09, *p* = 0.005). 45.5% of patients with sRIL had a positive surgical margin compared to 24.3% in patients with non-sRIL (*p* = 0.01). Patients with positive margins had larger treatment volumes and total doses (Table 1; Supplementary Table 2), which may have contributed to the sRIL. Prolonged RT duration may contribute to severe lymphopenia. Grade 3 to 4 lymphopenia occurred in 54.8% of patients at a median of the 5th week after RT started [20]. Patients with treatment duration >4 weeks had a 28.9% increase in the risk of grade 3–4 lymphopenia, compared to those with the treatment duration of 4 weeks or less (32.1% vs. 62.1%, *p* = 0.006). In the present study, the median duration of treatment in patients with sRIL is 6 weeks, while it was only 5.4 weeks in patients with non-sRIL.

Notably, the PTV was also significantly larger in patients with sRIL. Lymphopenia is likely caused by radiation exposure to lymphocytes circulating in the body. Larger PTV leads to a larger volume of organ exposed to radiation. Ellsworth et al. has pointed out that circulating lymphocytes should be considered an organ at risk during RT [19]. In NSCLC patients undergoing definitive RT, larger GTVs were correlated with lower lymphocyte nadirs [18]. Other factors that have been shown to be associated with lymphopenia include RT technique, baseline ALC, radiation of immune organ, as well as dosimetric parameters of lung and heart [14], [16], [18], [21], [22], [23]. Proton therapy reduced the risk of severe lymphopenia by 29% compared with photon therapy in esophageal cancer with neoadjuvant CRT (17.6% vs. 40.4%; OR 0.29, *p* < 0.0001) [24]. However, in the present study, we did not see an advantage using proton therapy, likely due to the small sample size and also patient heterogeneity in tumor location and treatment volumes.

Although we didn’t find a correlation of sRIL with heart dose, likely due to the fact that most of the postoperative treatment volumes lie superior to the base of the heart and therefore the relative heart dose was quite low, we did find lung dosimetric parameters to be correlated with severe lymphopenia. Total lung V5, V10, V20, and total lung mean dose were higher in patients with sRIL, with the latter being significantly correlated with sRIL on univariable and multivariable analyses. As showed in Supplementary Table 4, total lung mean dose was significantly correlated with sRIL (r = 0.212, *p* = 0.009). Therefore, only total lung mean dose was analyzed, considering lung V5, V10, V20 and total lung mean dose affect each other. This is in contrast to the Tang et al. study, which analyzed 711 patients receiving definitive CRT for NSCLC and found that lung V5 was significantly associated with lymphocyte nadirs [18]. The difference may be due to the variation of patients enrolled.

Another interesting consideration is whether a lower lymphocyte count due to surgery further contributed to the radiation-associated lymphopenia. A previous study demonstrated that reduced mean lymphocyte count was correlated with thoracic surgery (*p* < 0.0001) [25]. In a study investigating the association between postoperative lymphopenia and postoperative pneumonia, the lymphocyte nadir was 1.0 ± 0.5 × 10^9^/L which occurred on day 1 after the surgery; however, the lymphocytes increased gradually after that. Similarly, lymphocytes decreased to 1.1 ± 0.49 × 10^9^/L 3 days postoperatively but recovered on day 4 in advanced oral cancer treated with surgery. The reduction of lymphocytes caused by surgery is limited and typically recovers fairly quickly afterwards. This is not the same as the lymphopenia caused by radiotherapy. Several studies have indicated that lymphocyte counts decline exponentially during radiation, reaching nadir between 3 and 5 weeks from the start of radiotherapy [20], [24]. In addition, the lymphocyte counts are not restored fully for nearly half of the patients even 1–2 months after completing radiation therapy [26]. The difference may be due to different types of lymphocyte damage. The decrease in lymphocytes caused by surgery may be caused by the body’s acute stress response, while substantial damage from radiotherapy to lymphocytes contributed to lymphopenia. In our study, 13.5% (23/170) of patients experienced lymphopenia after surgery but before radiation; however, none of them undergone sRIL. Furthermore, only 7 of 23 patients developed sRIL during RT. Therefore, it seems that the contribution of surgery on lymphopenia is limited.

Previous studies have shown that the severity of lymphopenia is associated with clinical outcomes. Tang et al showed that higher lymphocyte nadirs were associated with prolonged OS (*p* = 0.01) and event-free survival (*p* < 0.001) [18]. Ladbury et al. retrospectively reviewed 117 patients with stage III NSCLC treated with definitive CRT and showed that grade ≥ 3 lymphopenia was correlated with higher estimated dose of radiation to immune cells (EDRIC, *p* = 0.004), while EDRIC was independently associated with OS (HR 1.17, *p* = 0.03) [23]. The results suggested that lymphopenia was correlated with poor survival. Similarly, Yellu et al. reviewed 151 NSCLC stage III treated with curative RT, which was stratified into standard dose (≤60 Gy) and high dose (>66 Gy) [27]. The high dose patients had lower ALC and higher mortality (*p* < 0.0001). Consistent with these studies, we also showed that sRIL is a poor prognostic factor for PFS and OS in stage I-III PORT patients.

This study has several limitations as a single-institution retrospective study with a relatively small sample size. We also had to widen the time period for which this data was collected due to the lack of diagnostic lab collections for patients undergoing PORT alone without chemotherapy. This is especially true in more recent years since only 8.8% (15/170) of patients between 2015 and 2017 had their blood drawn for complete blood count analysis. As a consequence, most of the patient data were collected before 2015, when fewer patients received proton radiotherapy. In addition, the inclusion of patients with stage I-II disease increased the rate of patients with positive surgical margins (31.2%) which confounded the relative impact of sRIL. Defining a separate subset analysis in the stage III disease was a way to better study the impact of sRIL after PORT.

In conclusion, we demonstrated that patients with NSCLC receiving PORT were vulnerable to sRIL. Severe radiation-induced lymphopenia was correlated with total lung mean dose and radiation fractionation numbers and associated with poorer survival outcomes. Future studies will need to address the effects of using more hypofractionated course of radiotherapy or advanced radiation delivery techniques like proton therapy to further reduce the risk of sRIL. These approaches are especially relevant in this era of immunotherapies as these agents are increasingly being incorporated sequentially with radiotherapy.

## Funding

This study was funded in part by the Shandong Cancer Hospital for WJ and the 10.13039/100000002National Institutes of Health through MD Anderson's Cancer Center Support Grant CA016672.

## Patient Consent Statement

This is a retrospective analysis of previous treatment data on IRB approved protocol RCR05-0967 which waives patient consent of chart review for clinical outcomes analysis in thoracic cancer patients.

## Declaration of Competing Interest

The authors declare that they have no known competing financial interests or personal relationships that could have appeared to influence the work reported in this paper.
