# Supplementary material for: Prognosis of severe lymphopenia after postoperative radiotherapy in non-small cell lung cancer: Results of a long-term follow up study
Source: Clin Transl Radiat Oncol. 2021 Mar 12;28:54–61. doi: 10.1016/j.ctro.2021.02.011 (PMC7985216; doi:10.1016/j.ctro.2021.02.011)

**Supplementary**

Table 1. Comparisons of radiation-related parameters between the sRIL and non-sRIL groups.

|  | sRIL (mean ± SD) | Non-sRIL (mean ± SD) | *p* value |
| --- | --- | --- | --- |
| Heart V5 | 40.2 ± 31.8 | 36.0 ± 28.1 | 0.416 |
| Heart V10 | 35.1 ± 30.5 | 29.1 ± 24.9 | 0.197 |
| Heart V20 | 25.9 ± 24.6 | 21.6 ± 21.3 | 0.272 |
| Heart V30 | 21.3 ± 21.9 | 16.8 ± 18.9 | 0.187 |
| Heart V40 | 16.3 ± 18.4 | 13.0 ± 15.1 | 0.240 |
| Heart V50 | 10.2 ± 12.9 | 7.5 ± 10.0 | 0.162 |
| Heart mDose | 1335.9 ± 1195.2 | 1130.5 ± 1060.0 | 0.284 |
| Total lung V5 | 44.9 ± 16.7 | 39.0 ± 16.9 | 0.045 |
| Total lung V10 | 34.0 ± 12.9 | 29.1 ± 13.4 | 0.036 |
| Total lung V15 | 26.9 ± 10.3 | 23.4 ± 10.9 | 0.066 |
| Total lung V20 | 23.2 ± 9.6 | 19.6 ± 9.0 | 0.026 |
| Total lung mDose | 1330.1 ± 486.2 | 1101.4 ± 487.3 | 0.008 |

Abbreviations: sRIL, severe radiation-induced lymphopenia; V5, organ volume receiving 5 Gy; mDose, mean radiation dose.

Table 2. Univariate and multivariate analyses for progression-free survival in the stage III patients.

| Characteristic | Univariable analysis | |  | Multivariable analysis | | | |
| --- | --- | --- | --- | --- | --- | --- | --- |
|  | HR (95% CI) | *p* value |  | HR (95% CI) | | | *p* value |
| Gender |  |  |  |  | | |  |
| Male vs. female | 1.7 (0.98 - 2.18) | 0.06 |  | |  |  | |
| Race |  |  |  |  | | |  |
| White vs. Non-white | 1.58 (0.96 - 2.62) | 0.07 |  |  | | |  |
| Age (≥ 60 vs. < 60) | 0.90 (0.60 - 1.34) | 0.61 |  |  | | |  |
| COPD | 1.46 (0.85 - 2.49) | 0.17 |  |  | | |  |
| Smoking^a^ | 1.81 (1.08 - 3.03) | 0.02 |  | 1.79 (1.07 - 3.00) | | | 0.026 |
| Tumor site |  |  |  |  | | |  |
| Right vs. Left | 1.03 (0.69 - 1.52) | 0.90 |  |  | | |  |
| Surgery type |  |  |  |  | | |  |
| Sublobar resection | Ref |  |  |  | | |  |
| Lobectomy | 0.63 (0.34 - 1.19) | 0.16 |  |  | | |  |
| Pneumonectomy | 0.83 (0.37 - 1.89) | 0.67 |  |  | | |  |
| Pathological type |  |  |  |  | | |  |
| NEU | Ref |  |  |  | | |  |
| ADC | 1.20 (0.67 - 2.14) | 0.54 |  |  | | |  |
| SCC | 1.95 (1.01 - 3.76) | 0.04 |  |  | | |  |
| Surgical margin |  |  |  |  | | |  |
| R1/2 vs. R0 | 1.34 (0.87 - 2.07) | 0.18 |  |  | | |  |
| LVI | 1.11 (0.74 - 1.66) | 0.62 |  |  | | |  |
| Tumor grade |  |  |  |  | | |  |
| Well | Ref |  |  |  | | |  |
| Moderate | 0.71 (0.34 - 1.45) | 0.34 |  |  | | |  |
| Poor | 1.13 (0.55 - 2.34) | 0.73 |  |  | | |  |
| pT stage |  |  |  |  | | |  |
| T3 - 4 vs. T1 - 2 | 1.58 (1.02 - 2.44) | 0.04 |  |  | | |  |
| pN stage |  |  |  |  | | |  |
| N2 - 3 vs. N1 - 2 | 0.54 (0.28 - 1.04) | 0.07 |  |  | | |  |
| pStage (IIIB vs. IIIA) | 1.41 (0.62 - 3.24) | 0.41 |  |  | | |  |
| Adjuvant chemo | 0.88 (0.59 - 1.31) | 0.52 |  |  | | |  |
| RT technique |  |  |  |  | | |  |
| Proton vs. Photon | 0.95 (0.48 - 1.88) | 0.88 |  |  | | |  |
| Lymphopenia |  |  |  |  | | |  |
| sRIL vs. non-sRIL | 1.64 (1.09 - 2.46) | 0.02 |  | 1.62 (1.08 - 2.44) | | | 0.021 |

Abbreviations: CardioDis, Cardiovascular disease; COPD, chronic obstructive pulmonary disease; Smoking^a^, Prior/Current vs. Never; NEU, neuroendocrine carcinoma; ADC, adenocarcinoma; SCC, squamous cell carcinoma; R0/R1/R2: complete resection, microscopic residual tumor, macroscopic residual tumor; LVI, lymphovascular invasion; pT/N stage, pathological tumor/node stage; sRIL, severe radiation-induced lymphopenia.

Table 3. Univariate and multivariate analyses for overall survival in the stage III patients.

| Characteristic | Univariable analysis | |  | Multivariable analysis | |
| --- | --- | --- | --- | --- | --- |
|  | HR (95% CI) | *p* |  | HR (95% CI) | *p* |
| Gender |  |  |  |  |  |
| Male vs. Female | 1.45 (0.96 - 2.18) | 0.08 |  |  |  |
| Race |  |  |  |  |  |
| White vs. Non-white | 1.77 (1.03 - 3.04) | 0.04 |  | 1.78 (1.03 - 3.07) | 0.037 |
| Age (≥ 60 vs. < 60) | 1.25 (0.82 - 1.92) | 0.30 |  |  |  |
| COPD | 1.32 (0.74 - 2.33) | 0.35 |  |  |  |
| Smoking^a^ | 2.10 (1.19 - 3.72) | 0.01 |  |  |  |
| Tumor site |  |  |  |  |  |
| Right vs. Left | 1.17 (0.77 - 1.77) | 0.46 |  |  |  |
| Surgery type |  |  |  |  |  |
| Sublobar resection | Ref |  |  |  |  |
| Lobectomy | 0.89 (0.46 - 1.73) | 0.73 |  |  |  |
| Pneumonectomy | 0.97 (0.40 - 2.33) | 0.94 |  |  |  |
| Patho type |  |  |  |  |  |
| NEU | Ref |  |  | Ref |  |
| ADC | 1.56 (0.79 - 3.04) | 0.20 |  | 1.69 (0.86 - 3.31) | 0.128 |
| SCC | 2.76 (1.32 - 5.76) | 0.01 |  | 3.15 (1.50 - 6.61) | 0.002 |
| Surgical margin |  |  |  |  |  |
| R1/2 vs. R0 | 1.33 (0.84 - 2.09) | 0.22 |  |  |  |
| LVI | 1.05 (0.68 - 1.61) | 0.84 |  |  |  |
| Tumor grade |  |  |  |  |  |
| Well | Ref |  |  |  |  |
| Moderate | 0.70 (0.33 - 1.51) | 0.37 |  |  |  |
| Poor | 1.14 (0.53 - 2.44) | 0.74 |  |  |  |
| pT stage |  |  |  |  |  |
| T3 - 4 vs. T1 - 2 | 1.57 (0.99 - 2.48) | 0.05 |  |  |  |
| pN stage |  |  |  |  |  |
| N2 - 3 vs. N0 - 1 | 0.42 (0.21 - 0.82) | 0.01 |  |  |  |
| pStage (IIIB vs. IIIA) | 1.76 (0.76 - 4.05) | 0.18 |  |  |  |
| Adjuvant chemo | 0.73 (0.48 - 1.10) | 0.13 |  |  |  |
| RT technique |  |  |  |  |  |
| Proton vs. Photon | 0.78 (0.34 - 1.78) | 0.55 |  |  |  |
| Lymphopenia |  |  |  |  |  |
| sRIL vs. non-sRIL | 1.77 (1.16 - 2.68) | 0.01 |  | 1.88 (1.23 - 2.88) | 0.004 |

Abbreviations: CardioDis, Cardiovascular disease; COPD, chronic obstructive pulmonary disease; Smoking^a^, Prior/Current vs. Never; NEU, neuroendocrine carcinoma; ADC, adenocarcinoma; SCC, squamous cell carcinoma; R0/R1/R2: complete resection, microscopic residual tumor, macroscopic residual tumor; LVI, lymphovascular invasion; pT/N stage, pathological tumor/node stage; POCT, postoperative chemotherapy. sRIL, severe radiation-induced lymphopenia.

Table 4. Spearman correlation coefficients between the percentage of lung radiation dose and severe radiation-induced lymphopenia.

| Lung dosimetry | sRIL | |
| --- | --- | --- |
|  | r | *p* |
| Lung V5 | 0.152 | 0.062 |
| Lung V10 | 0.181 | 0.026 |
| Lung V15 | 0.163 | 0.046 |
| Lung V20 | 0.185 | 0.023 |
| Total lung mDose | 0.212 | 0.009 |

Abbreviations: sRIL, severe radiation-induced lymphopenia. V5, organ volume receiving 5 Gy; mDose, mean radiation dose.

Figure 1. Overall survival and progression-free survival of 170 patients.


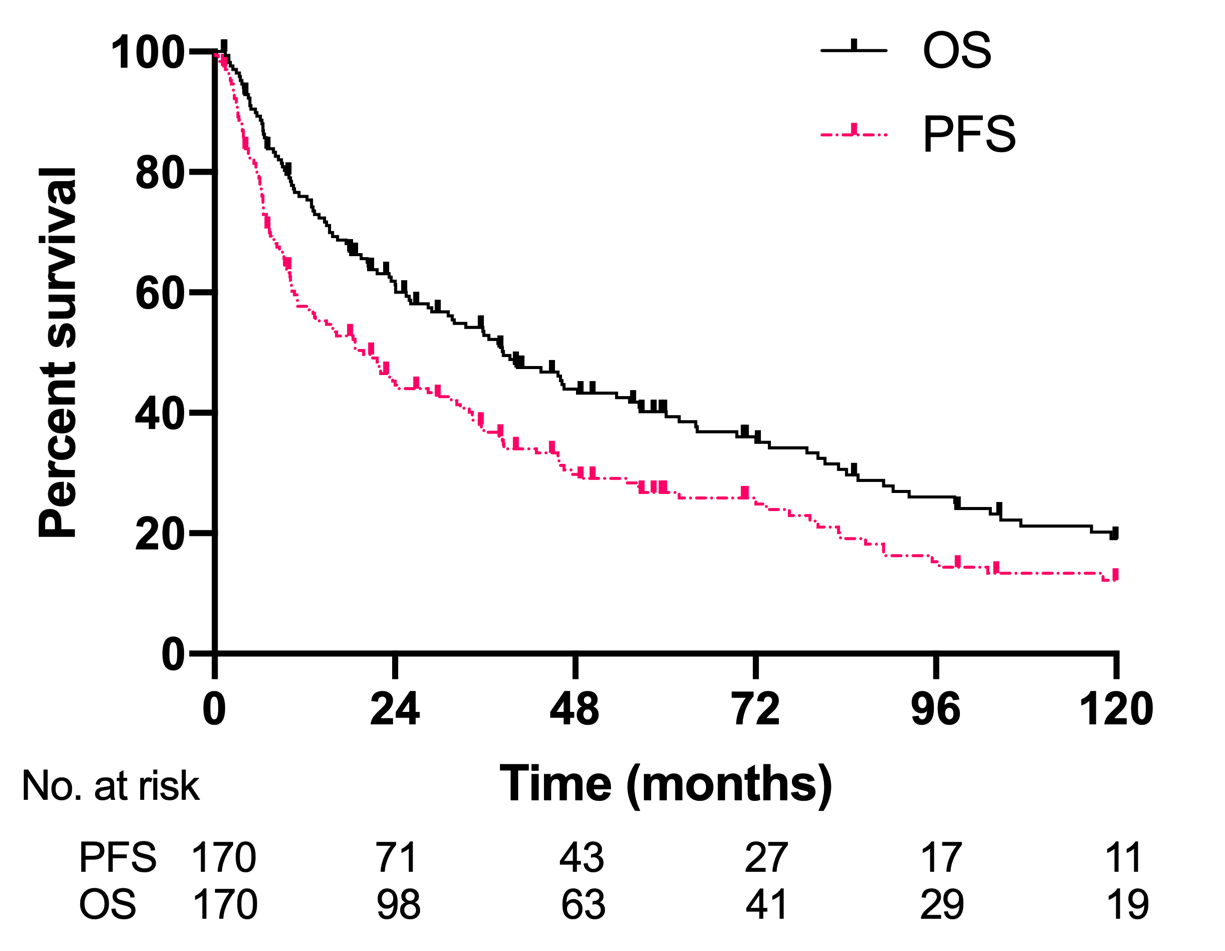

Supplement: Supplementary data 1 [file mmc1.docx]
